# Chemotherapy-induced macrophage CXCL7 expression drives tumor chemoresistance via the STAT1/PHGDH-serine metabolism axis and SAM paracrine feedback to M2 polarization

**DOI:** 10.1038/s41419-025-07712-y

**Published:** 2025-05-14

**Authors:** Shuguang Liu, Hui Gong, Peihang Li, Jiahao Hu, Yixuan Li, Rou Xu, Junchao Cai, Shuqi Wang, Jiayi Cai, Hongmei Ma, Xirong Mi, Yifan Li, Qingbo Zhou, Qiming Zhou, Weiqiang Yang, Riqing Li, Libing Song, Lishan Fang

**Affiliations:** 1 Department of pathology, The Eighth Affiliated Hospital, Sun Yat-Sun University, Shenzhen, China; 2 Shenzhen Nanshan People’s Hospital, Shenzhen, China; 3 Medical Research Center, The Eighth Affiliated Hospital, Sun Yat-Sun University, Shenzhen, China; 4 Department of Immunology, Zhongshan School of Medicine, Sun Yat-Sen University, Guangzhou, China; 5 Department of Microbiology, Zhongshan School of Medicine, Sun Yat-Sen University, Guangzhou, China; 6 Department of Pharmacology, Zhongshan School of Medicine, Sun Yat-Sen University, Guangzhou, China; 7 Shenzhen University Medical School, Shenzhen, Guangdong China; 8 Department of Biochemistry, Zhongshan School of Medicine, Sun Yat-sen University, Guangzhou, China; 9 Department of Otolaryngology, Peking University Shenzhen Hospital, Shenzhen, China; 10 Shenzhen Inspection and Testing Center of Agricultural Product Quality and Safety, Shenzhen, China; 11 Department of Experimental Research, State Key Laboratory of Oncology in South China, Collaborative Innovation Center for Cancer Medicine, Sun Yat-sen University Cancer Center, Guangzhou, China

**Keywords:** Cancer microenvironment, Cancer metabolism

## Abstract

Chemotherapy resistance in colorectal cancer (CRC) remains a major obstacle in clinical oncology. Analysis of clinical specimens from chemotherapy-resistant patients revealed elevated CXCL7 expression in tumor-associated macrophages (TAMs). Through integrated in vitro and in vivo studies, we demonstrated that chemotherapy induces tumor cell-macrophage crosstalk, leading to CXCL7 upregulation in TAMs. Using a co-culture system, we observed that CXCL7+ macrophages confer chemoresistance to CRC cells. Mechanistic investigations revealed that CXCL7 activates the CXCR2 receptor on tumor cells, triggering interferon signaling and promoting serine metabolism through STAT1-dependent transcriptional upregulation of phosphoglycerate dehydrogenase (PHGDH), the key enzyme in serine biosynthesis. This metabolic reprogramming enhances the paracrine secretion of S-adenosyl methionine (SAM), which drives chemotherapy resistance. Furthermore, CXCL7-mediated the paracrine secretion of SAM in tumor cells, which in turn promotes M2 macrophage polarization and sustains CXCL7 expression in TAMs. Our findings reveal that a CXCL7-SAM feedback loop between tumor cells and macrophages establishes a chemoresistant niche. This interaction represents a promising therapeutic target for overcoming chemoresistance in CRC.

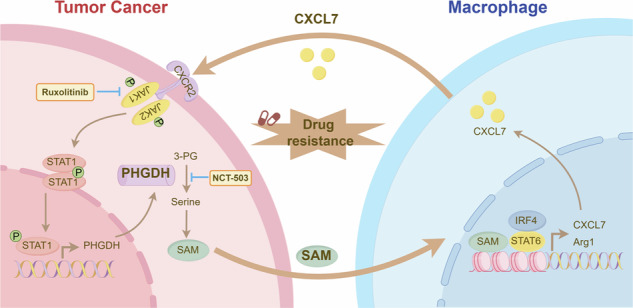

## Introduction

Chemotherapy resistance remains a significant obstacle in the effective treatment of colorectal cancer (CRC), resulting in poor clinical outcomes [1–3]. The tumor microenvironment (TME) plays a crucial role in this resistance, with tumor-associated macrophages (TAMs) [4], particularly those polarized into the M2 phenotype, being key contributors [5]. M2 macrophages, which are associated with immune suppression and tumor progression, secrete factors that support tumor growth and repair, thereby fostering an environment conducive to resistance [6]. However, the specific signaling pathways through which M2 macrophages contribute to resistance are not fully understood, and their interactions with other microenvironmental factors warrant further investigation.

CXCL7, a chemokine also known as platelet-derived growth factor (PDGF) is encoded by the gene pro-platelet basic protein (PPBP) and has been implicated in various cancers due to its role in promoting tumor progression and metastasis [7–9]. In CRC, CXCL7 levels are associated with tumor aggressiveness and poor prognosis [10]. Recent studies suggest that CXCL7 can influence the TME by modulating immune cell behavior and enhancing inflammation, which may contribute to chemoresistance [11]. However, the specific mechanisms by which CXCL7 affects CRC chemoresistance, especially through interactions with TAMs and metabolic pathways, have not yet been fully elucidated.

Serine metabolism is another crucial factor in chemotherapy resistance [12]. Serine is an amino acid that supports several metabolic pathways essential for tumor growth and survival [13]. The serine synthesis pathway, particularly the enzyme phosphoglycerate dehydrogenase (PHGDH), plays a pivotal role in this process [14, 15]. Elevated serine metabolism has been linked to increased chemoresistance, as it contributes to the cellular stress response and the biosynthesis of key molecules required for tumor cell survival [16, 17]. Despite these associations, the detailed role of serine metabolism in CRC chemoresistance requires further exploration.

Our study explored the role of CXCL7 in CRC chemoresistance, focusing on its effects on serine metabolism and TAMs. We discovered that chemotherapy-induced CXCL7 expression in TAMs contributes to resistance by activating the CXCL7/CXCR2 axis in CRC cells, which in turn enhances serine metabolism through the upregulation of PHGDH. Additionally, CXCL7 triggers the interferon signaling pathway, where STAT1 enhances the transcription of PHGDH. This process increases serine metabolism and SAM production, which promotes M2 macrophage polarization and creates a feedback loop that reinforces chemoresistance. These insights into the CXCL7/CXCR2-STAT1-PHGDH axis and SAM-induced M2 polarization offer new perspectives for targeting CRC chemoresistance.

## Materials and Methods

### Cell culture

The human colorectal cancer cell lines HT29, HCT116, and CT26 were obtained from the Shanghai Institutes of Biological Sciences Cell Bank (Shanghai, China). These cells were maintained in DMEM medium (Gibco, C11995500BT) supplemented with 10% fetal bovine serum (FBS; Gibco, 10270106) and 1% penicillin/streptomycin (Invitrogen). The human monocytic cell line THP-1 (ATCC) was cultured in RPMI-1640 medium (Gibco, C11875500BT) containing 10% FBS and 1% penicillin/streptomycin. All cell lines were authenticated by short tandem repeat (STR) profiling at the Forensic Medicine Department Laboratory of Sun Yat-Sen University (Guangzhou, China) and confirmed to be free of mycoplasma contamination.

### Differential expression analysis

RNA-seq data from colorectal cancer (CRC) samples were retrieved from The Cancer Genome Atlas (TCGA) database. Differential expression analysis was performed using the DESeq2 package in R (version 4.0.5, R Foundation for Statistical Computing). Significantly upregulated genes in chemotherapy-resistant CRC samples treated with 5-fluorouracil (5-FU) and oxaliplatin were identified using a cutoff of |log_2_FC|> 1 and *p*-value < 0.05.

### Gene Set Enrichment Analysis (GSEA)

Gene Set Enrichment Analysis (GSEA) is a computational method used to determine whether a predefined set of genes exhibits statistically significant differences between two phenotypes. In this study, GSEA was conducted using two groups stratified by the median CXCL7 mRNA expression in TCGA data. The analysis was performed using GSEA version 2.0.9 (Broad Institute, http://www.broadinstitute.org/gsea/).

### Immunohistochemistry (IHC)

Paraffin-embedded CRC specimens were obtained from the Pathology Department of the Eighth Affiliated Hospital of Sun Yat-sen University. Prior patient consent and approval from the Institutional Research Ethics Committee of the Eighth Affiliated Hospital of Sun Yat-sen University were obtained for the use of these clinical materials for research purposes. IHC assay was performed and quantitatively analyzed as previously described [18]. Primary antibodies used were anti-CXCL7 (1:200, Affinity, #DF6695), anti-CD11b (1:1000, Proteintech, 66519-1-lg), anti-CD68 (1:2000, Proteintech, 28058-1-AP), anti-CD206 (1:2000, Proteintech, 18704-1-AP), and anti-PHGDH (1:500, Proteintech, 14719-1-AP). Diaminobenzidine (DAB) was used for visualization, and hematoxylin (Sigma-Aldrich, 03971) was used for counterstaining. Two independent pathologists evaluated staining intensity, while data analysis was conducted by two independent investigators blinded to the sample groups.

### Single-cell RNA sequencing analysis (scRNA-seq)

scRNA-seq data of CRC tissues were retrieved from the TISCH2 database. CXCL7 expression was analyzed across various cell populations using the Seurat package (R version 4.0.5). Macrophage subtypes were identified based on canonical marker expression, and CXCL7 expression levels were assessed across clusters.

### Co-culture experiments with macrophage

THP-1 cells were differentiated into macrophages through a 48-hour treatment with 100 ng/mL phorbol 12-myristate 13-acetate (PMA, MedChemExpress, HY-18739). Macrophages were co-cultured with medium only or colorectal cancer cell HCT116 in a transwell system (Corning, 3422) and treated with individually or in combination of 5-FU (5 µM, GBCBIO, 0597) and oxaliplatin (5 µM, MedChemExpress, HY-17371) for 48 hours. Macrophage polarization markers were subsequently analyzed.

### Flow cytometry and apoptosis assay

Cell apoptosis was measured by the Annexin V-FITC/PI Apoptosis Detection Kit (Servicebio, G1511) following a 48-hour treatment with 5-FU and oxaliplatin. CRC cell lines were harvested with EDTA-free trypsin solution (Servicebio, G4002), washed with PBS two times, centrifuged at 500 × *g* for 5 minutes each time, resuspended in binding buffer, and incubated with 5 μL/test Annexin V-FITC and 5 μL/test PI for 10 min at room temperature in the dark. The cells were analyzed by flow cytometry (BD Biosciences) within an hour, and data were processed using FlowJo software (version 10.0, BD Biosciences).

### TUNEL assay

Apoptosis was evaluated using the TUNEL assay kit (Servicebio, G1501) according to the manufacturer’s instructions. HCT116 and HT29 cells treated with 5-FU and oxaliplatin for 48 hours were fixed in 4% paraformaldehyde. Apoptotic cells were visualized and imaged using a fluorescence microscope (Zeiss, Germany).

### Cell viability assays

HCT116 and HT29 cells were seeded in 12-well plates (Corning, 3513) and cultured for 24 hours to allow adherence. Following treatment with 5-FU and oxaliplatin for 48 hours, cells were fixed with 100% methanol and stained with 0.5% crystal violet solution (Beyotime, C0121). Colony numbers were quantified using ImageJ software (NIH), and all experiments were performed in triplicate.

### Multi-color immunohistochemistry (mIHC)

Immunohistochemical staining of tumor samples from both chemotherapy-sensitive and chemotherapy-resistant patients was conducted using a four-color IHC kit (Absin, China). The slides were first subjected to deparaffinization with xylene, rehydrated, and then washed in EDTA-citrate buffer for antigen retrieval by microwave treatment. Endogenous peroxidase activity was inhibited by applying an antibody diluent/blocking reagent (PerkinElmer, USA). Primary antibodies targeting CD68 (1:500, Proteintech, 28058-1-AP), CXCL7 (1:500, Affinity, #DF6695), and CK (1:500, Affinity, #DF3072) were applied and incubated at room temperature for one hour. The slides were then incubated with Polymer HRP Ms+Rb at 37 °C for 10 minutes. Signal amplification was achieved using TSA fluorescent dyes (TSA520, TSA570, and TSA650) diluted in amplification buffer, with 10-minute incubations at room temperature. Between staining rounds, antigen-antibody complexes were stripped by microwave treatment in 0.05% Tris-EDTA buffer (pH 9.0). Images were captured using a confocal laser scanning microscope (Zeiss Microscopy, USA).

### Animal experiments

All animal experiments were conducted following ethical guidelines and were approved by the Institutional Animal Care and Use Committee (IACUC) of the Eighth Affiliated Hospital of Sun Yat-sen University. BALB/c nude mice and immunocompetent BALB/c mice (6 weeks old, male, Shenzhen TOP Biotechnology Co. China) were used for in vivo studies.

For the colorectal cancer xenograft Model, HT29 cells stably overexpressing CXCL7 or control cells were injected subcutaneously into the right flank of BALB/c nude mice. Mice were treated with 5-FU (50 mg/kg) and oxaliplatin (2.5 mg/kg), administered intraperitoneally twice a week for 3 weeks. We performed subcutaneous co-injection of CXCL7+macrophages or CXCL7-macrophages with tumor cell lines into BALB/c nude mice, followed by combination chemotherapy with 5-FU and oxaliplatin, with regular tumor size measurements.

In the syngeneic transplant model, BALB/c mice were intravenously administered clodronate liposomes (200 μl per mouse; YEASEN, Shanghai, China) for macrophage depletion prior to CT26 cell injection and combination chemotherapy. CT26 murine colorectal cancer cells were subcutaneously injected into BALB/c mice, followed by treatment with anti-CXCL7 antibody (20 μg/mouse, R&D Systems, AF793) or isotype control antibodies, with tumor volume monitoring and subsequent tumor collection for apoptosis assays and IHC analysis.

For AOM/DSS Model, intestinal polyps were induced in immunocompetent BALB/c mice using azoxymethane (AOM, 10 mg/kg, Sigma-Aldrich, A5486) and 2% dextran sulfate sodium (DSS, Yeasen, 60316ES) in drinking water for 5 days followed by three additional cycles, with subsequent treatment using either anti-CXCL7 antibody or isotype control antibodies, combined with 5-FU and oxaliplatin. Intestinal polyp quantification and tissue collection for apoptosis and macrophage polarization analysis using IHC and TUNEL assays. At least five mice per group were used to ensure adequate power, and each mouse of different weights was randomly allocated. Primary tumors were not performed in a blinded manner.

### RNA extraction and RT-qPCR

Total RNA from CRC cells was extracted using the Total RNA Kit II (GBCBIO, R4105) following the manufacturer’s instructions. RNA samples were then reverse transcribed into complementary DNA (cDNA) using standard procedures. Quantitative real-time PCR (qRT-PCR) was performed on a Roche LightCycler 96 system (Roche Diagnostics) with SYBR Green I dye (Molecular Probes, Accurate Biology, AG11701) [19]. The sequences of primer are listed as following:

**Table:** 

Primer name	Sequence
Human PPBP primers	5’-CCCTTCCTGTAACAGTGCGA-3’(forward)
	5’-AGCAGTCAGCAGCAATGACA-3’(reverse)
Human PHGDH primers	5’-TGCAAATCTGCGGAAAGTGC-3’(forward)
	5’-GATGACATCAGCGGTCACCT-3’(reverse)
Human STAT6 primers	5’-CCTTGGAGAACAGCATTCCTGG-3’(forward)
	5’-GCACTTCTCCTCTGTGACAGAC-3’(reverse)
Human IRF4 primers	5’-GAACGAGGAGAAGAGCATCTTCC-3’(forward)
	5’-CGATGCCTTCTCGGAACTTTCC-3’(reverse)
Human β-actin primers	5’-GAGACCTTCAACACCCCAGC-3’(forward)
	5’-GATAGCACAGCCTGGATAGCA-3’(reverse)

### RNA sequencing and metabolomic analysis

Treated tumor cells were quickly collected and frozen in liquid nitrogen and then were stored at 80 °C. The RNA extraction, library construction, sequencing, and the transcriptome analysis were completed by Gene Denovo Biotechnology Company (Guangzhou, China). Differentially expressed genes and pathways were conducted using DESeq2 (R package) and Gene Set Enrichment Analysis (GSEA). Metabolomic profiling was performed using liquid chromatography-mass spectrometry (LC-MS, Thermo Fisher Scientific) to evaluate alterations in serine metabolism.

### Western blot analysis

Total protein was extracted from cell lines using RIPA lysis buffer (Beyotime, P0013B), and protein concentration was quantified using a BCA assay kit (Beyotime, P0011). Protein samples were separated by SDS-PAGE, transferred to PVDF membranes, and blocked with 5% non-fat milk. Primary antibodies used included anti-PHGDH (1:2000, Proteintech, 14719-1-AP), anti-PSAT1 (1:1000, Affinity, #DF12132), anti-PSPH (1:1000, Affinity, #DF12711), anti-STAT1 (1:1000, Affinity, #AF6300), anti-p-STAT1 (1:1000, Affinity, #AF3300), anti-JAK1 (1:1000, Affinity, #AF5012), anti-p-JAK1 (1:1000, Affinity, #AF2012), anti-IRF9 (1:1000, Affinity, #DF8179) and anti-GAPDH (1:10000, Affinity, #AF7021). Blots were incubated with HRP-conjugated secondary antibodies (1:5000, Proteintech, SA00001-1, SA00001-2), and protein bands were detected using enhanced chemiluminescence (GBCBIO, G3308).

### S-adenosyl Methionine (SAM) ELISA Assay

Intracellular SAM levels were measured using an ELISA kit (BYabscience, BY-JZF0309) according to the manufacturer’s protocol. Tumor cells overexpressing CXCL7 or control cells were treated with PHGDH inhibitor for 48 hours. Subsequently, cell culture supernatants were collected for SAM quantification.

### CHIP-qPCR

ChIP-qPCR was performed using a ChIP kit (Bersinbio, #Bes5001) according to the manufacturer’s instructions. Briefly, cells were cross-linked, washed, and centrifuged before lysis in buffer containing protease inhibitors. Chromatin extracts were sonicated, and equal aliquots of supernatant were subjected to immunoprecipitation overnight at 4 °C with either anti-STAT1 antibody or control IgG antibody, coupled to protein G magnetic beads. After sequential washing, reverse cross-linking, and DNA purification, the immunoprecipitated DNA was analyzed by real-time quantitative PCR using SYBR GreenER qPCR SuperMix.

### Luciferase Reporter Assays

The PHGDH promoter region was cloned into the pGL3-basic vector (Promega), and luciferase activity was measured using the Dual-Luciferase Reporter Assay System (Beyotime, RG005) according to the protocol as described previously. Briefly, CRC cells (10^4^ cells/well) were seeded in 96-well plates in triplicate and allowed to settle for 24 h. Plasmid transfection was performed using Lipofectamine 3000 transfection reagent (Thermo Fisher Scientific, L3000001) according to the manufacturer’s instructions.

### Statistical analysis

Statistical analysis was performed using SPSS version 24.0 (SPSS Inc., Chicago, IL, USA). Sample size was determined by power analysis to achieve a minimum effect size of 0.5 with a *P* value of <0.05, and all sample sizes were appropriate for the assumption of normal distribution. All data were presented as mean ± standard deviation (SD). Statistical analyses were conducted using Student’s t-test or one-way ANOVA with post-hoc analysis where appropriate. Correlations between CXCL7 expression and clinical parameters were assessed using the chi-square test. Kaplan-Meier methodology was used to evaluate survival probabilities, and log-rank test was used to compare survival differences on univariate analysis. Multivariate statistical analysis was performed using a Cox regression model. *P* < 0.05 was considered to indicate statistical significance. Variance within each group of data was estimated and was similar between the compared groups.

## Results

### CXCL7 is upregulate in chemotherapy-resistant tumors

**Fig. 1:**
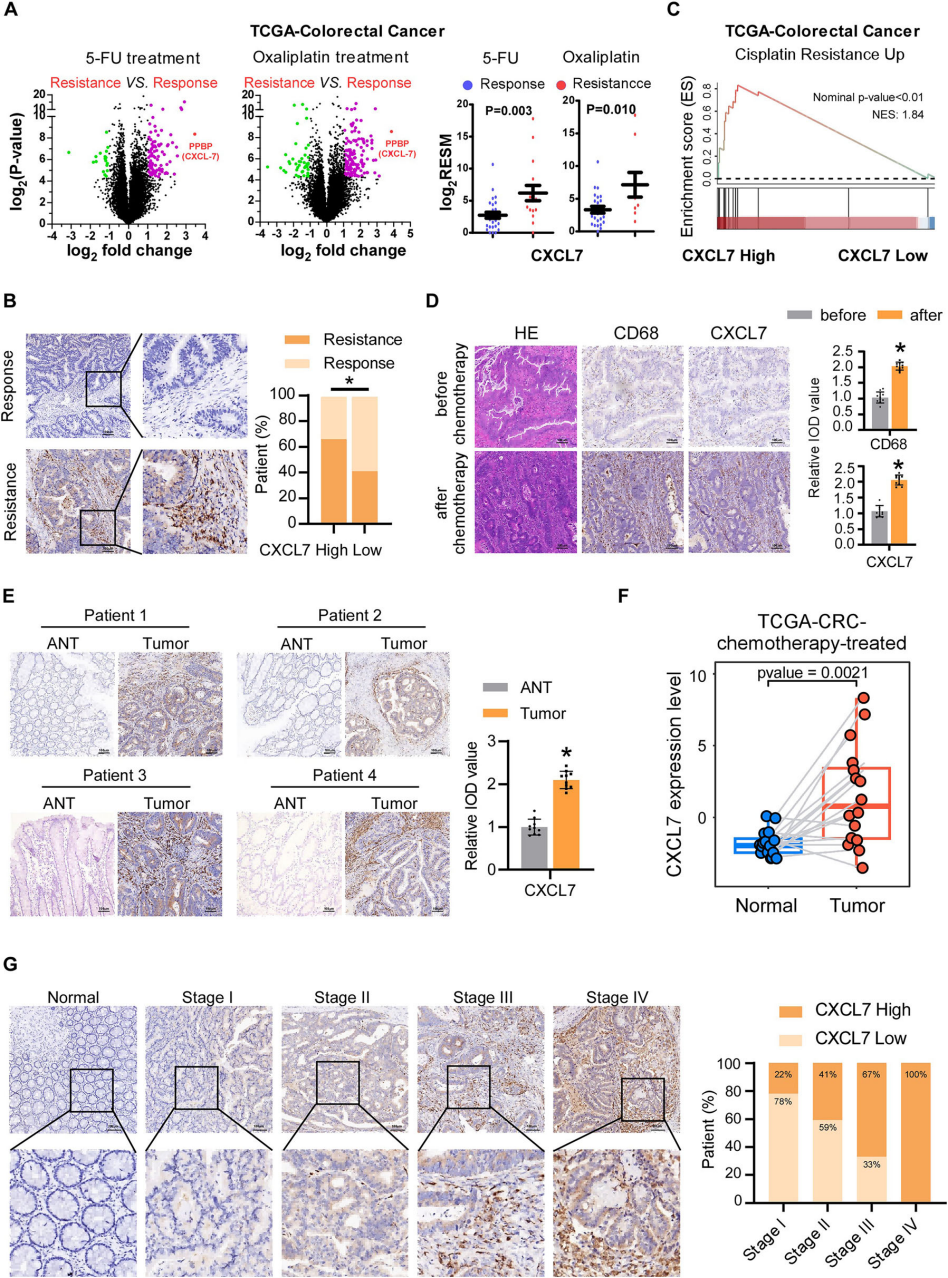
CXCL7 upregulation in chemotherapy-resistant colorectal cancer. **A** Differential gene expression profiling between chemosensitive and chemoresistant colorectal tumors (TCGA cohort) treated with 5-FU/oxaliplatin combination therapy. **B** Representative immunohistochemical (IHC) staining of CXCL7 in chemotherapy-resistant versus chemotherapy-sensitive CRC specimens (Scale bar, 100 μm). **C** Gene Set Enrichment Analysis (GSEA) demonstrating cisplatin resistance pathway activation in CXCL7-high versus CXCL7-low expression subgroups. **D** Comparative histopathological analysis using H&E staining and IHC evaluation of CXCL7/CD68 expression in paired pre- and post-chemotherapy CRC specimens from individual patients (Scale bar, 100 μm). **E** CXCL7 protein localization by IHC in chemotherapy-treated CRC lesions versus matched adjacent normal mucosa (Scale bar, 100 μm). **F** Quantitative comparison of CXCL7 expression levels between chemotherapeutically treated malignant tissues and non-neoplastic colorectal tissues. **G** Stage-dependent IHC expression patterns of CXCL7 across colorectal cancer progression (Scale bar, 100 μm). All data are presented as mean ± SD. *P < 0.05; n.s.not significant.

To identify key genes involved in chemotherapy resistance in colorectal cancer (CRC), we performed a differential expression analysis using publicly available data from The Cancer Genome Atlas (TCGA). Our analysis revealed that the chemokine CXCL7 (also known as PPBP) is significantly upregulated in chemotherapy-resistant CRC tumors treated with 5-FU and oxaliplatin (Fig. 1A), which was further confirmed by IHC in our clinical samples (Fig. 1B). GSEA analysis further confirmed a positive correlation between CXCL7 expression and cisplatin resistance (Fig. 1C). To determine whether CXCL7 is derived from primary or acquired resistance, we examined the expression of CXCL7 and CD68 in tumor tissues before and after chemotherapy from the same patient. The results revealed that CXCL7 expression was upregulated and co-expressed with CD68 in macrophages following chemotherapy (Fig. 1D). Additionally, CXCL7 expression was significantly increased in chemotherapy-treated CRC tumor samples compared with the corresponding adjacent non-tumor tissues, which was further confirmed by TCGA (Fig. 1E, F).

In a cohort of 60 paraffin-embedded CRC specimens, we assessed the correlation between CXCL7 expression levels and clinical characteristics. As shown in Table 1, CXCL7 expression was correlated with clinical stage (p = 0.029), T stage (*p* = 0.024), N stage (*p* = 0.023), MLH1 deficiency (*p* = 0.010) and RNF43 mutation (*p* = 0.037), but was not correlated with age, gender, histological differentiation or MSH2. Notably, the expression of CXCL7 was significantly positively correlated with the expression of a marker gene of microsatellite instability, MLH1 deficiency. In our previous research, we established the role of RNF43 mutations in the tumor microenvironment of patients with microsatellite instability [20]. In this context, we further observed a significant correlation between CXCL7 expression and RNF43 mutations. The proportion of RNF43 mutations in patients with high CXCL7 expression was 36.67%, which was significantly greater than the 13.33% observed in patients with low CXCL7 expression. As colorectal cancer progresses, the sensitivity to chemotherapeutic agents significantly decreases in patients with advanced stages of the disease [21]. As shown in Fig. 1G, CXCL7 expression was nearly undetectable in normal colorectal tissues, whereas its expression was significantly elevated in advanced-stage (III and IV) CRC tissues. Taken together, these data suggest a relationship between CXCL7 expression and the tumor microenvironment, promoting chemotherapy resistance in CRC.

**Table 1:** Correlation between the clinicopathological features and expression of CXCL7.

Title		CXCL7 group(%)		Total	χ^2^	*P* value
		Low	High			
Gender	female	13(43.3)	11(36.7)	24	0.278	0.598
	male	17(56.7)	19(63.3)	36		
Age	<65	19(63.3)	13(43.3)	32	2.411	0.121
	>=65	11(36.7)	17(56.7)	28		
Clinical stage	Stage I	7(23.3)	2(6.7)	9	9.037	0.029*
	Stage II	16(53.3)	11(36.7)	27		
	Stage III	7(23.3)	14(46.7)	21		
	Stage IV	0(0.0)	3(10.0)	3		
T classifcation	T1	1(3.3)	2(6.7)	3	9.433	0.024*
	T2	11(36.7)	3(10.0)	14		
	T3	17(56.7)	18(60.0)	35		
	T4	1(3.3)	7(23.3)	8		
N classifcation	N0	23(76.7)	13(43.3)	36	7.521	0.023*
	N1	5(16.7)	9(30.0)	14		
	N2	2(6.7)	8(26.7)	10		
Histological diferentiation	Poor	3(12.0)	8(28.57)	11	3.137	0.208
	Moderate	21(84.0)	20(71.43)	41		
	Well	1(4.0)	0(0.0)	1		
MLH1	Negative	4(13.3)	13(43.3)	17	6.648	0.010**
	Positive	26(86.7)	17(56.7)	43		
MSH2	Negative	6(20.0)	2(6.7)	8	2.308	0.129
	Positive	24(80.0)	28(93.3)	52		
RNF43	Wildtype	26(86.7)	19(63.3)	45	4.356	0.037*
	Mutant	4(13.3)	11(36.7)	15		

* *p* < 0.05 ** *p* < 0.01.

### Macrophage-derived CXCL7 promotes chemotherapy resistance in tumor cells

**Fig. 2:**
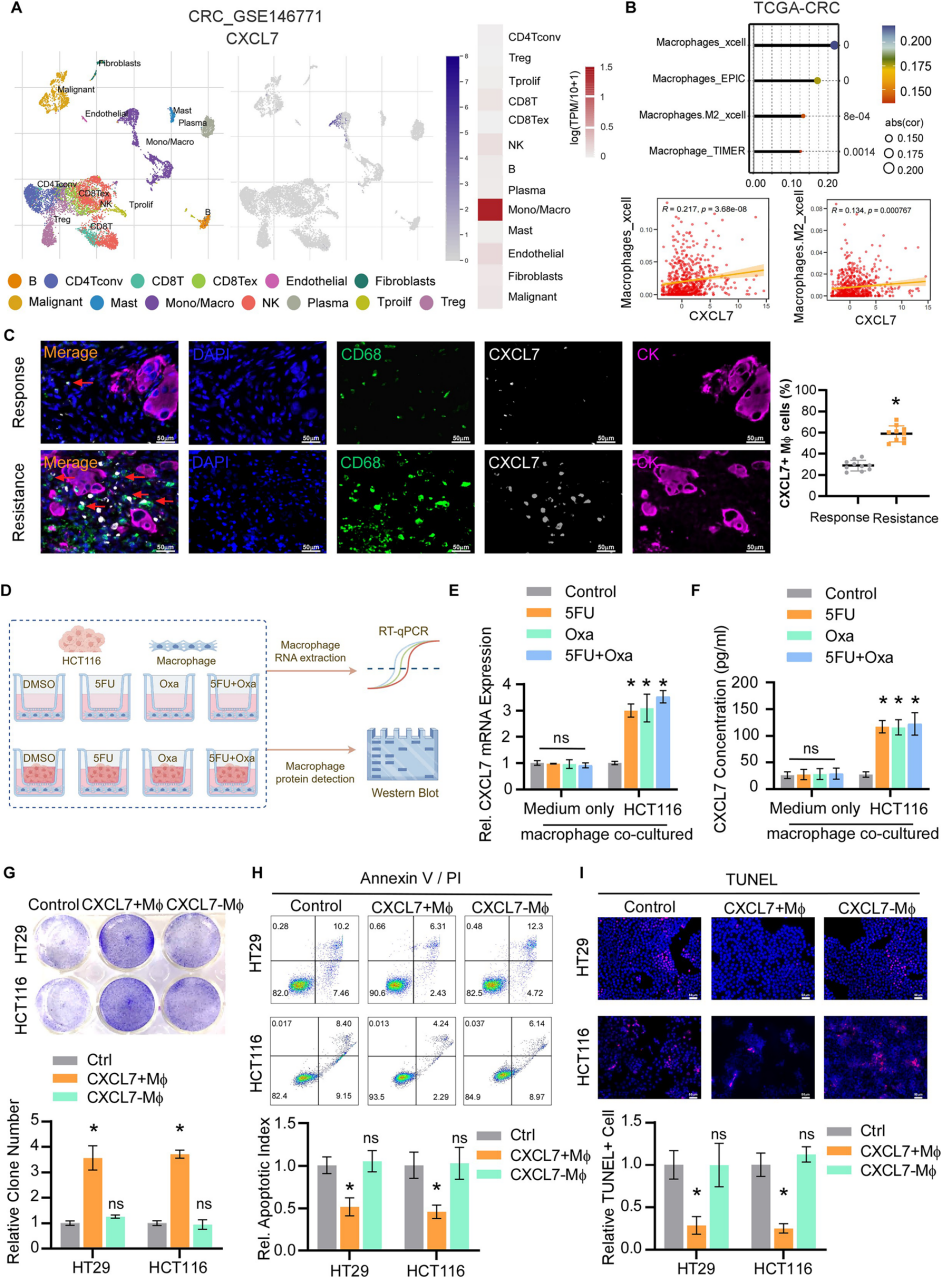
Macrophage-derived CXCL7 promotes chemoresistance in colorectal cancer cells. **A** CXCL7-associated single-cell analysis of CRC_GSE146771 using the TISCH2 database. **B** Correlation analysis of CXCL7 with macrophage infiltration via TCGA database. **C** Left: mIHC staining of chemotherapy-sensitive versus resistant CRC tissues showing CK+ (purple), CXCL7+ (white), and CD68+ macrophages (green), with DAPI nuclear staining (blue) (scale bar, 50 μm). Right: CXCL7+ macrophage proportions in chemotherapy-sensitive versus resistant patients. **D** Schematic of tumor cell-macrophage co-culture system. **E** CXCL7 mRNA and **F** CXCL7 protein levels in macrophages co-cultured with HCT116 cells ± 5-FU (5 μM), oxaliplatin (5 μM), or combination. **F** The expression of CXCL7 in chemotherapy-treated colorectal cancer tissues compared with non-tumor tissues was analyzed. **G** Representative images and quantification for colony formation of CRC cells with CXCL7 + Mø versus CXCL7- Mø under 5-FU/oxaliplatin treatment. **H** Annexin V/PI apoptosis rates and (I) TUNEL+ cells (green) in CRC cells co-cultured with CXCL7 + Mø versus CXCL7- Mø under chemotherapy (scale bar, 50 μm). All data are presented as mean ± SD. *P < 0.05; n.s.not significant.

Given our experimental observation that CXCL7 is expressed primarily in TAMs, we further conducted a single-cell analysis of tumor tissues to explore the source of CXCL7 expression using the TISCH2 database. The results further revealed that CXCL7 expression was predominantly enriched in macrophages (Fig. 2A). Furthermore, CXCL7 expression was significantly correlated with macrophages in the TCGA-CRC dataset (Fig. 2B). The mIHC analyses revealed colocalization of CXCL7 with the macrophage marker CD68 in colorectal cancer clinical samples. The proportion of CXCL7-positive macrophages was significantly increased in chemotherapy-resistant patients (Fig. 2C). In a co-culture system of tumor cells and macrophages, chemotherapy-induced tumor cells stimulated CXCL7 expression in macrophages, whereas chemotherapy drugs alone failed to directly upregulate CXCL7 expression in macrophages (Fig. 2D–F). These results indicate that chemotherapy enhances the crosstalk between tumor cells and macrophages, leading to increased secretion of CXCL7 by macrophages.

To explore the role of macrophage-derived CXCL7 in tumor cells, we treated tumor cell lines with 5-FU and oxaliplatin with either CXCL7-negative macrophages (CXCL7-Mø) or CXCL7-positive macrophages (CXCL7 + Mø). Annexin V apoptosis assays and TUNEL assays demonstrated that CXCL7 + Mø significantly reduced chemotherapy-induced apoptosis in CRC cells, leading to increased colony formation (Fig. 2G–I). These findings suggest that macrophage-derived CXCL7 mediates tumor cell resistance to 5-FU and oxaliplatin.

### Macrophage-derived CXCL7 mediates tumor chemotherapy resistance in vivo

**Fig. 3:**
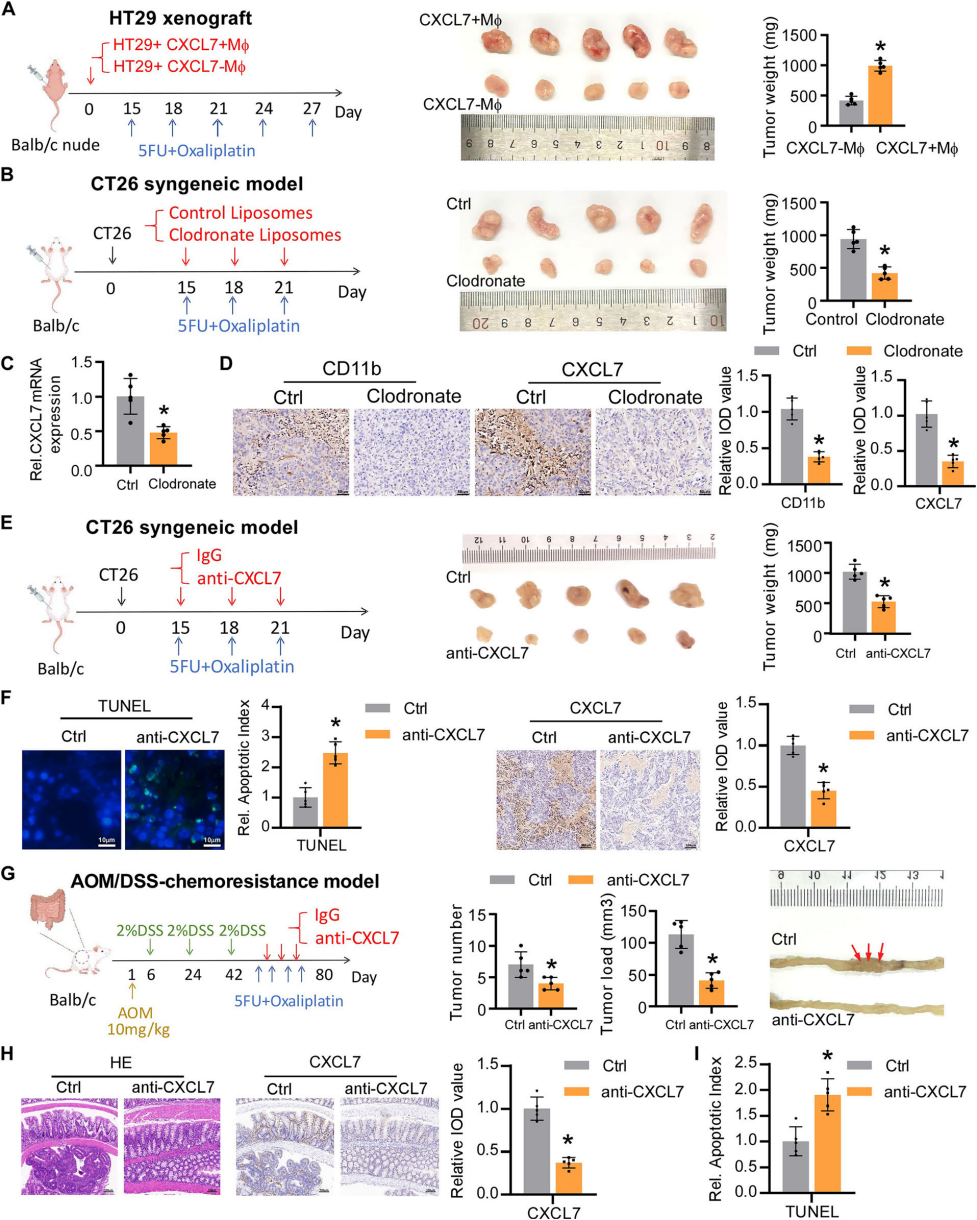
CXCL7 Mediates Tumor Chemotherapy Resistance in vivo. **A** Representative images and body weights of mice injected with CXCL7 + Mø or CXCL7- Mø under 5-FU/oxaliplatin treatment (*n* = 5 mice per group). **B** CT26 tumor growth in immunocompetent BALB/c mice treated with control or macrophage depletion (*n* = 5 mice per group). **C** CXCL7 expression levels in macrophage-intact versus macrophage-depleted tumors analyzed by RT-qPCR. **D** IHC staining of CD11b+ cells and CXCL7+ expression in control versus clodronate-treated tumors (scale bar, 50 μm). **E** CT26 tumor progression in mice treated with isotype control or CXCL7-neutralizing antibodies (*n* = 5 mice per group). **F** TUNEL-positive cells and representative IHC images of CXCL7 in the tumors obtained from mice treated with isotype control or CXCL7 neutralizing antibodies (scale bar for TUNEL, 10 μm; scale bar for IHC, 100 μm). **G** Intestinal polyp quantification (number/size) and representative images from AOM/DSS mice treated with control or CXCL7-neutralizing antibodies (scale bar, 100 μm). **H** HE and IHC detection of CXCL7 expression across different groups of mice, as indicated (scale bar, 100 μm). **I** TUNEL+ apoptotic cell counts in AOM/DSS mice with/without CXCL7 antibody treatment. All data are presented as mean ± SD. *P < 0.05; n.s.not significant.

To evaluate the impact of macrophage-secreted CXCL7 on tumor resistance, we subcutaneously co-injected CXCL7 + Mø or CXCL7-Mø with HT29 cell lines in BALB/c nude mice. The results revealed that tumors in the CXCL7 + Mø group exhibited greater resistance to chemotherapy (Fig. 3A). Since CXCR2 is the receptor for CXCL7 and is significantly expressed on colorectal cancer tumor cells [22], we aimed to verify the role of CXCL7 in mediating chemoresistance via CXCR2. The results showed that CXCL7 overexpression enhanced tumor resistance to chemotherapy, whereas the addition of the CXCR2 inhibitor reversed this effect (Fig. S1A). These results indicate that the CXCL7/CXCR2 signaling axis mediates cancer cell resistance to 5-FU and oxaliplatin in tumor cells.

In the CT26 Balb/c syngeneic model treated with a combination of 5-FU and oxaliplatin, we observed a decrease in CXCL7 expression in macrophages following macrophage depletion with clodronate liposomes (Fig. 3B–D). Macrophage depletion or neutralization of CXCL7 led to tumor shrinkage and enhanced drug sensitivity (Fig. 3B, E). TUNEL staining also confirmed that CXCL7 inhibited cell apoptosis.

Consistantly, in a mouse model induced by azoxymethane/dextran sulfate sodium (AOM/DSS), we investigated whether CXCL7-neutralizing antibodies could reduce resistance during combination treatment with 5-FU and oxaliplatin. Compared with those in control mice, those treated with CXCL7-neutralizing antibodies exhibited a significant reduction in both the number of intestinal polyps (Fig. 3G) and chemotherapy-induced apoptosis (Fig. 3H, I).

Interestingly, although neutralizing antibodies against CXCL7 do not directly affect the expression of target genes, immunohistochemistry (IHC) of mouse tumor sections showed that CXCL7 neutralizing antibodies suppressed CXCL7 expression (Figs. 3F, H), suggesting the existence of a positive feedback loop that sustains CXCL7 expression within the tumor microenvironment.

### CXCL7 promotes chemotherapy resistance through the serine metabolism pathway

**Fig. 4:**
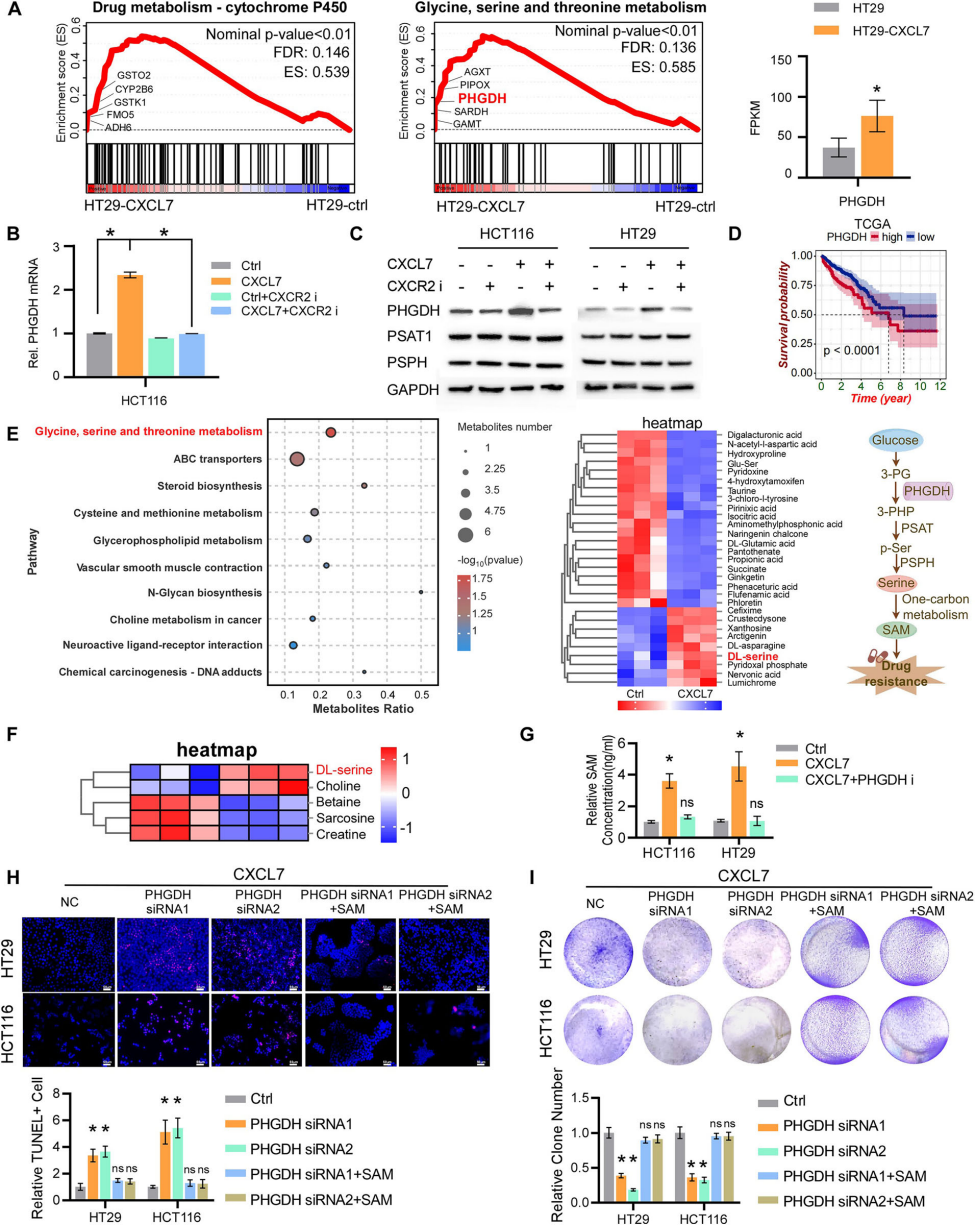
CXCL7 upregulates PHGDH and activates serine metabolism in cancer cells. **A** Left: GSEA of enriched pathways in differentially expressed genes (DEGs) between HT29-control and HT29-CXCL7 groups. Right: Comparative analysis of PHGDH FPKM values between groups. **B** The expression level of PHGDH in control and CXCL7-overexpressing cells with or without the CXCR2 inhibitor, as determined by RT-qPCR. **C** Western blot detection of serine biosynthesis enzymes (PHGDH, PSAT1, PSPH).GAPDH was used as the loading control. **D** Kaplan‒Meier analysis of overall survival in TCGA-CRC patients stratified by PHGDH expression. **E** Left: Metabolite enrichment pathway bubble plot. Middle: Heatmap of differential metabolites. Right: Schematic of serine/one-carbon metabolism. **F** Heatmap visualization of DEGs in glycine, serine, and threonine metabolism pathways. **G** ELISA analysis of SAM levels in CXCL7-overexpressing cells and PHGDH-inhibited cells. **H** TUNEL assays of CXCL7-overexpressing cells transfected with NC-siRNA or PHGDH-siRNA, with or without SAM treatment(scale bar, 50 μm). **I** The drug sensitivity of CXCL7-overexpressing cells transfected with NC-siRNA or PHGDH-siRNA, with or without SAM treatment. All data are presented as mean ± SD. *P < 0.05; n.s.not significant.

To investigate the mechanism underlying CXCL7-induced chemoresistance, we performed RNA sequencing (RNA-seq) on control and CXCL7-treated tumor cells. Pathway analysis revealed that CXCL7 upregulated the signaling pathways involved in drug resistance and serine metabolism (Fig. S2A, Fig. 4A). Notably, phosphoglycerate dehydrogenase (PHGDH), the rate-limiting enzyme in the serine synthesis pathway, was significantly upregulated by CXCL7 (Fig. 4A). Consistent with the RNA-seq results, RT-qPCR also showed the upregulation of PHGDH at the mRNA level when CXCL7 was upregulated (Fig. 4B). This effect was further validated at the protein level using Western blotting, which revealed that CXCL7 overexpression increased the protein level of PHGDH, whereas this effect was blocked by a CXCR2 inhibitor. Additionally, CXCL7 overexpression had no effect on the expression of PSAT or PSPH (Fig. 4C). Additionally, analysis of the TCGA-CRC database confirmed that elevated PHGDH expression is significantly correlated with poor prognosis in colorectal cancer patients (Fig. 4D). Metabolomic analysis further confirmed that CXCL7 activation enhances serine metabolism and its downstream one-carbon metabolism pathway in tumor cells (Fig. 4E, F). Previous studies have demonstrated that increased serine metabolism promotes one-carbon metabolism [23], leading to the production of S-adenosyl methionine (SAM), which contributes to tumor drug resistance [24–26]. ELISA assays confirmed that CXCL7 overexpression elevated SAM levels, and this effect was blocked by PHGDH inhibition (Fig. 4G), suggesting that CXCL7-induced serine metabolism in tumor cells mediates SAM secretion.

To further validate the role of PHGDH in CXCL7-mediated chemoresistance, we conducted functional assays by knocking down PHGDH in CXCL7-overexpressing CRC cells. PHGDH knockdown significantly increased chemotherapy-induced apoptosis and enhanced drug sensitivity. Notably, supplementation with SAM partially rescued this effect (Fig. 4H, I). These results suggest that CXCL7 promotes chemoresistance by enhancing serine metabolism and the upregulation of SAM.

### CXCL7 promotes serine metabolism and chemotherapy resistance through the STAT1-mediated transcription of PHGDH

**Fig. 5:**
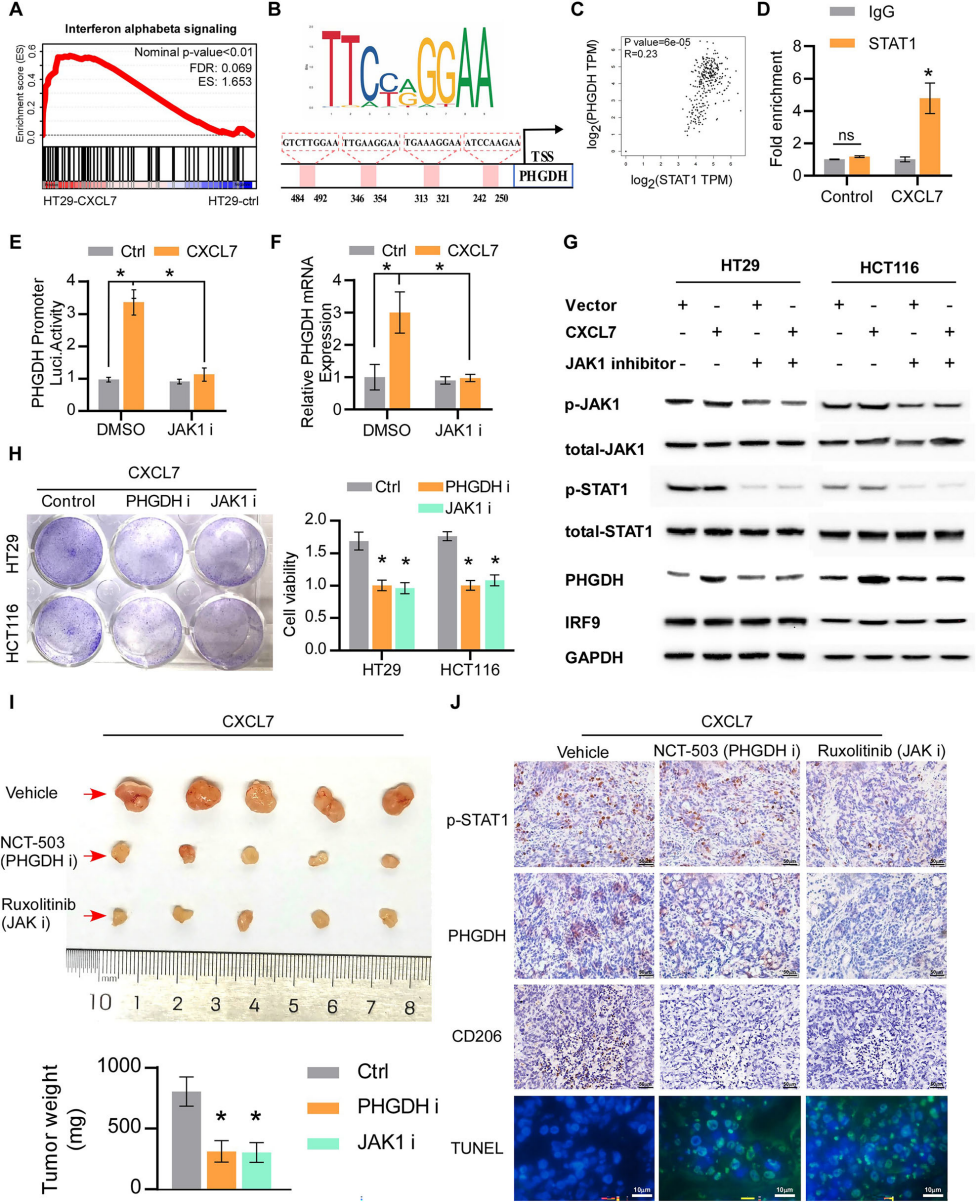
STAT1 mediates CXCL7-induced PHGDH transcription and chemotherapy resistance. **A** GSEA analysis illustrating the correlation between CXCL7 and the interferon signaling pathway. **B** Predicted STAT1-binding sequences in the PHGDH promoter region using the JASPAR database. **C** The correlationship between STAT1 and PHGDH mRNA expression. **D** ChIP-qPCR results showing STAT1 enrichment at the PHGDH promoter. **E** Luciferase reporter assay results demonstrating PHGDH promoter activity in response to CXCL7 treatment with or without JAK1 inhibition. **F** Effects of JAK1 inhibition on PHGDH mRNA expression following CXCL7 treatment. **G** Western blot analysis of p-JAK1, JAK1, p-STAT1, STAT1, PHGDH, and IRF9 protein levels in cells treated with CXCL7 with or without JAK1 inhibition. **H** Cell viability assessment for cell treatment with CXCL7, following PHGDH inhibition or JAK1 inhibition. **I** Effects of PHGDH or JAK1 inhibition on tumor growth in mice with CXCL7-overexpressing cell. **J** IHC analysis of p-STAT1, PHGDH, and CD206 expression, along with TUNEL staining for apoptosis, in CXCL7-overexpressing tumors treated with PHGDH or JAK1 inhibitor (scale bar for IHC, 50 μm; scale bar for TUNEL, 10 μm). All data are presented as mean ± SD. *P < 0.05; n.s.not significant.

To elucidate the molecular mechanism by which CXCL7 upregulates PHGDH, we analyzed CXCL7-associated signaling pathways and found that CXCL7 activated the interferon signaling pathway (Fig. 5A). STAT1, a key transcription factor in this pathway, has been identified as a regulator of PHGDH transcription, and database analysis further confirmed the correlation between STAT1 activity and PHGDH expression (Fig. 5B, C). ChIP-qPCR revealed that CXCL7 significantly increased STAT1 occupancy at the promoter of PHGDH (Fig. 5D). In addition, luciferase reporter assays showed that CXCL7 overexpression increased the activity of the PHGDH promoter reporter gene, an effect that was reversed by the JAK1 inhibitor (which inhibits STAT1 expression), indicating that CXCL7 upregulates PHGDH expression through STAT1 (Fig. 5E). As demonstrated in Fig. 5F, G, the JAK1 inhibitor blocked the upregulation of PHGDH mRNA and protein levels induced by CXCL7 overexpression. Additionally, both in vitro and in vivo experiments demonstrated that the PHGDH inhibitor or JAK1 inhibitor enhanced the chemotherapy sensitivity of CXCL7-overexpressing tumors (Fig. 5H–J), indicating that CXCL7 upregulates PHGDH via STAT1, thereby activating serine synthesis and mediating tumor resistance to chemotherapy. Notably, both a PHGDH inhibitor and a JAK1 inhibitor reduced the expression of CD206, a marker of M2 macrophage polarization. This suggests that the STAT1/serine metabolism pathway in tumor cells plays a role in inhibiting macrophage M2 polarization.

### Tumor cell serine metabolism promotes macrophage M2 polarization and sustained CXCL7 expression through SAM paracrine signaling

**Fig. 6:**
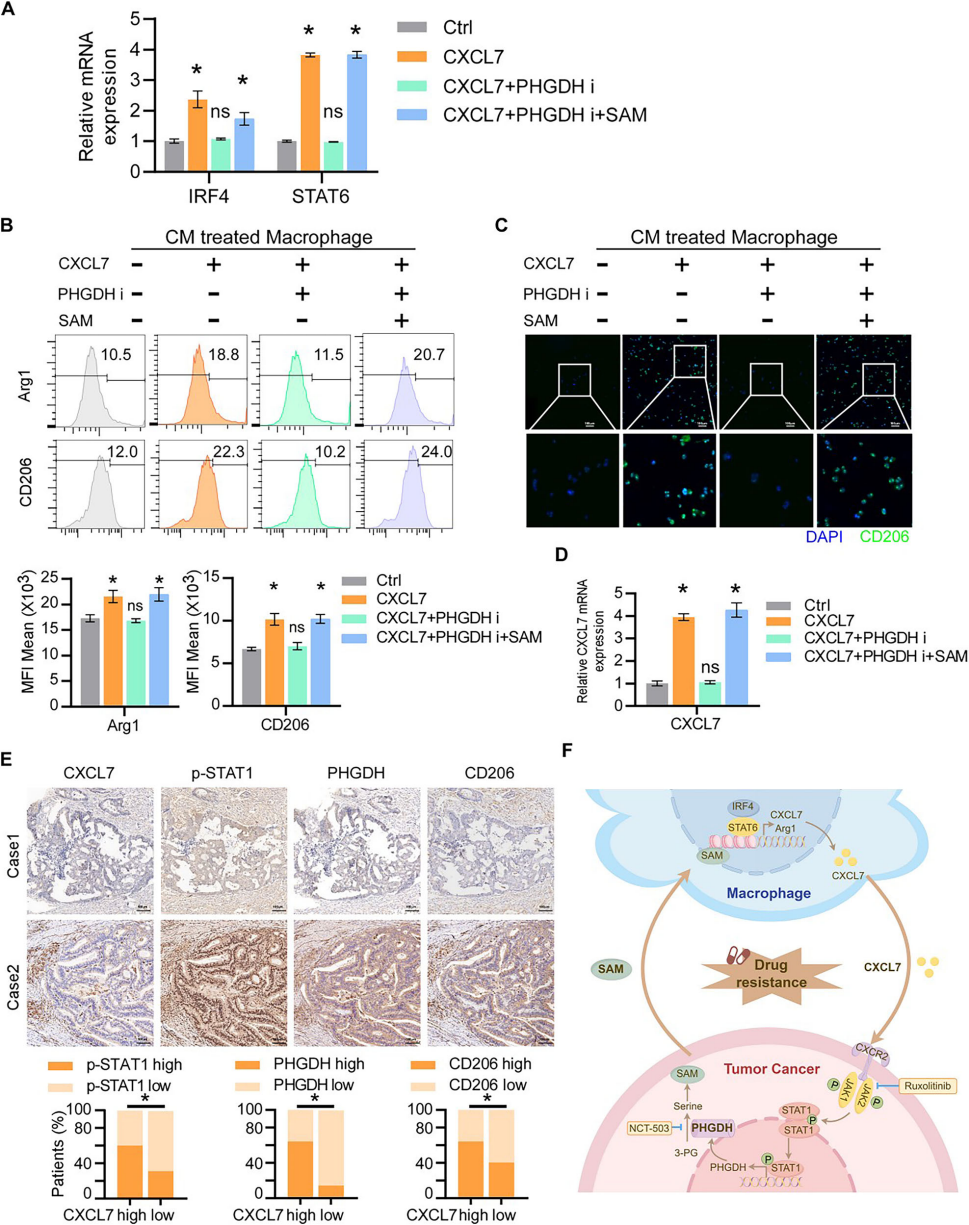
Serine metabolism promotes M2 macrophage polarization through SAM paracrine signaling. **A** Expression levels of IRF4 and STAT6 in macrophages treated with conditioned medium derived from indicated tumor cells. **B** Flow cytometry analysis of M2 macrophage marker expression in macrophages treated with the indicated conditioned medium. **C** Immunofluorescence staining showing CD206 expression in macrophages treated with the indicated conditioned medium (scale bar, 100 μm). **D** CXCL7 mRNA expression levels in macrophages exposed to conditioned medium from cancer cells treated with a PHGDH inhibitor, subjected to SAM rescue. **E** IHC staining of CRC samples showing the correlation between CXCL7 expression and PHGDH, p-STAT1, and CD206. Representative images of low and high CXCL7 expression cases are displayed, along with the percentage distribution of CRC samples exhibiting low or high CXCL7 expression relative to PHGDH, p-STAT1, and CD206 levels (scale bar, 100 μm). **F** Schematic representation of the correlation between CXCL7 expression and serine metabolism-related pathways in CRC, highlighting its role in M2 macrophage polarization and a potential feedback loop within the tumor microenvironment. All data are presented as mean ± SD. *P < 0.05; n.s.not significant.

Given the observed effect of sustained CXCL7 overexpression on M2 macrophage polarization in vivo, we further explored the molecular mechanism by which tumor cell serine metabolism influences macrophages. Previous studies have shown that SAM epigenetically modifies macrophages by inhibiting the transcriptional activity of IRF4 and STAT6, leading to the upregulation of arginase-1 expression and promoting M2 polarization [27]. To investigate this mechanism, we analyzed macrophages treated with conditioned medium from CXCL7-overexpressing tumor cells. We observed a significant increase in IRF4 and STAT6 mRNA levels in macrophages, an effect that was abolished by PHGDH inhibition and restored by SAM supplementation, confirming that CXCL7 regulates macrophage M2 polarization via PHGDH-mediated serine and SAM metabolism (Fig. 6A). Additionally, the expression of the M2 macrophage markers Arginase-1 and CD206 was elevated in the conditioned medium from CXCL7-overexpressing cells. However, PHGDH inhibition suppressed this effect, whereas SAM supplementation restored Arginase-1 and CD206 expression (Fig. 6B, C). Interestingly, inhibition of PHGDH significantly reduced CXCL7 mRNA levels in macrophages, whereas treatment with SAM restored these levels (Fig. 6D). These findings indicate that CXCL7-mediated serine metabolism in tumor cells regulates M2 polarization and CXCL7 expression in macrophages through SAM paracrine signaling.

### Clinical correlation of CXCL7 expression with serine metabolism and M2 macrophage polarization in CRC

Finally, we examined the clinical relevance of CXCL7 expression in relation to serine metabolism and macrophage polarization in colorectal cancer specimens. IHC analysis revealed a significant positive correlation between CXCL7 levels and the expression of p-STAT1 (*P* < 0.01), PHGDH (*P* < 0.01) and CD206 (*P* < 0.01) in CRC samples (*n* = 60; Fig. 6E).

## Discussion

Our study advances the understanding of CRC chemoresistance by revealing the pivotal role of CXCL7 and its mechanisms of action. Several key findings from our research provide new insights into this complex process:

### The role of CXCL7 secretion by macrophages in modulating tumor cell behavior

Our study revealed that CXCL7 is a key chemokine secreted by tumor-associated macrophages (TAMs) that promotes chemoresistance in CRC by activating the CXCR2 receptor on tumor cells. These findings underscore the pivotal role of TAMs in driving tumor progression and therapy resistance. Similarly, Sui *et al*. demonstrated that in lung adenocarcinoma, TAM-secreted S100A9 enhances tumor cell survival and resistance to current therapies by modulating inflammation and immune responses [28]. Additionally, a recent study highlighted the role of ERK-CXCL7 signaling in IFNα-induced BST2+ TAMs, facilitating immunosuppression and tumor growth in pancreatic cancer [11]. Collectively, these findings emphasize the significant role of TAM-secreted factors in the development of resistance across various cancer types. Targeting TAMs and their specific secreted factors, such as CXCL7 in CRC and S100A9 in lung adenocarcinoma, could therefore offer a strategy to intervene in resistance mechanisms tailored to different tumor types.

### The activation of PHGDH and serine metabolism promotes chemoresistance through the interferon signaling pathway

We demonstrated that CXCL7 activates the interferon signaling pathway in CRC cells through STAT1, leading to increased serine metabolism and contributing to chemoresistance. Recent studies have highlighted the role of PHGDH modifications in promoting serine synthesis and their potential as therapeutic targets in cancer. For example, Wang et al. [15] explored how arginine methylation of PHGDH enhances serine synthesis in hepatocellular carcinoma, whereas Zhang et al. reported that monoubiquitination of PHGDH increases its activity and contributes to cancer progression in CRC [29]. In contrast, our study links CXCL7 to both interferon signaling and PHGDH, revealing how CXCL7-induced metabolic reprogramming drives chemoresistance in CRC. These findings not only enrich the understanding of the role of PHGDH in serine metabolism but also emphasizes the critical importance of metabolic alterations in CRC chemoresistance.

### Feedback loop involving SAM and macrophage polarization

Importantly, our study revealed that CXCL7-induced increases in serine metabolism lead to increased production of SAM. SAM is then released into the TME, where it promotes M2 macrophage polarization and further upregulates CXCL7 expression in macrophages. This establishes a self-reinforcing feedback loop that further exacerbates chemoresistance. This feedback mechanism is supported by previous research indicating that SAM can influence macrophage polarization and contribute to cancer progression [27]. Our study highlights how this feedback loop, which is mediated by CXCL7 and SAM, integrates serine metabolism and macrophage polarization to increase CRC chemoresistance. The role of SAM in macrophage polarization and its impact on chemotherapy resistance underscore the complex interplay between metabolic pathways and immune cells functions in the tumor microenvironment.

In summary, our findings demonstrate that chemotherapy induces tumor cell-macrophage crosstalk, leading to CXCL7 upregulation in TAMs, which activates the CXCR2 receptor on tumor cells. This activation triggers STAT1-dependent transcriptional upregulation of PHGDH, enhancing serine metabolism and leading to the paracrine secretion of SAM, thereby promoting chemoresistance. Additionally, CXCL7-mediated SAM secretion from tumor cells drives M2 macrophage polarization and sustains CXCL7 expression in TAMs, establishing a self-reinforcing feedback loop between tumor cells and macrophages. This interplay highlights a critical mechanism underlying the chemoresistant niche in colorectal cancer (Fig. 6F).

## Supplementary information

Supplemental

western blots

## Data Availability

All RNA-seq data have been deposited in the National Center for Biotechnology Information Sequence Read Archive (SRA) database with BioProject accession code PRJNA1165170 (SRA study: SRR30792557, SRR30792558, SRR30792559, SRR30792560, SRR30792561, SRR30792562).
