# Supplementary material for: Chemotherapy-induced macrophage CXCL7 expression drives tumor chemoresistance via the STAT1/PHGDH-serine metabolism axis and SAM paracrine feedback to M2 polarization
Source: Cell Death Dis. 2025 May 14;16(1):379. doi: 10.1038/s41419-025-07712-y (PMC12078479; doi:10.1038/s41419-025-07712-y)
Supplement: Supplementary file 1 — Supplemental [file 41419_2025_7712_MOESM1_ESM.docx]

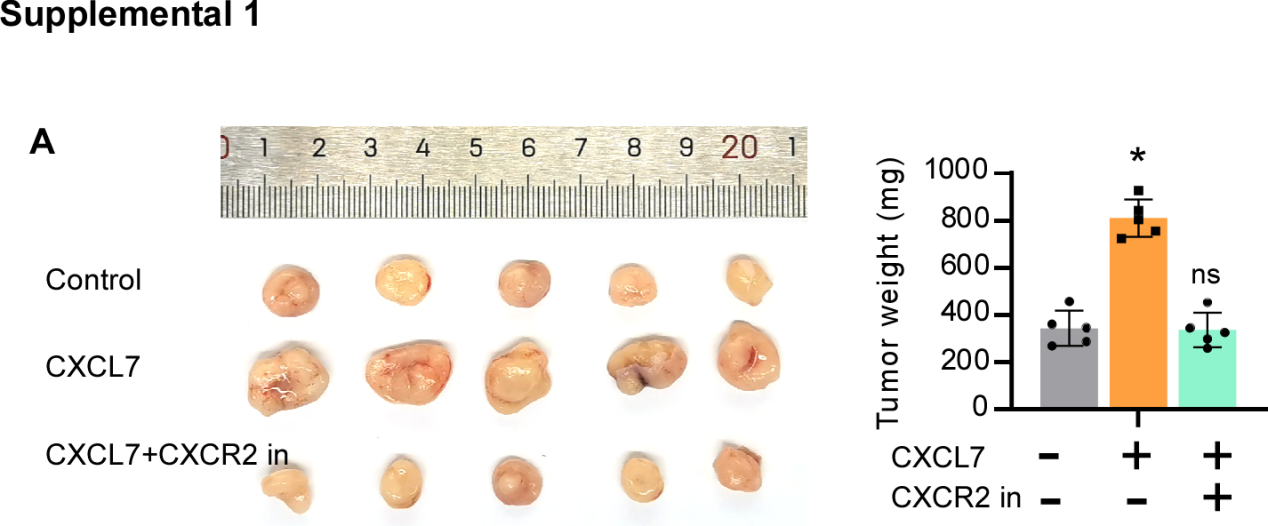


(A) Representative pictures (left) and weight (right) of indicated xenograft tumors in response to 5-FU and Oxaliplatin via intraperitoneal injection twice a week for 3 weeks (n=5 mice per group).


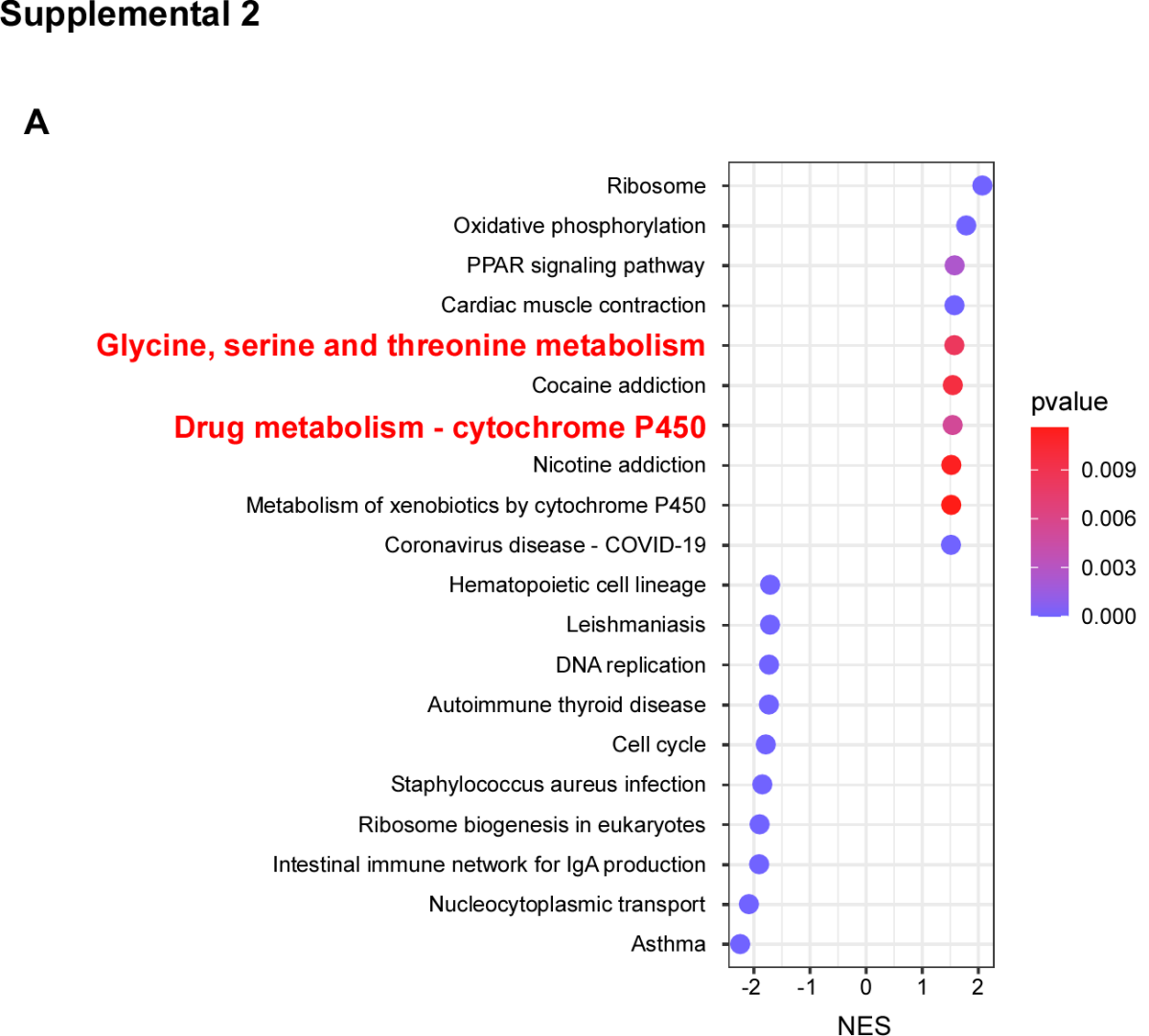


(A) The GSEA analysis of the enriched pathways for DEGs between the HT29 control group and the HT29-CXCL7 group is shown.
