# Supplementary figures and images for: Chemotherapy-induced macrophage CXCL7 expression drives tumor chemoresistance via the STAT1/PHGDH-serine metabolism axis and SAM paracrine feedback to M2 polarization
Source: Cell Death Dis. 2025 May 14;16(1):379. doi: 10.1038/s41419-025-07712-y (PMC12078479; doi:10.1038/s41419-025-07712-y)

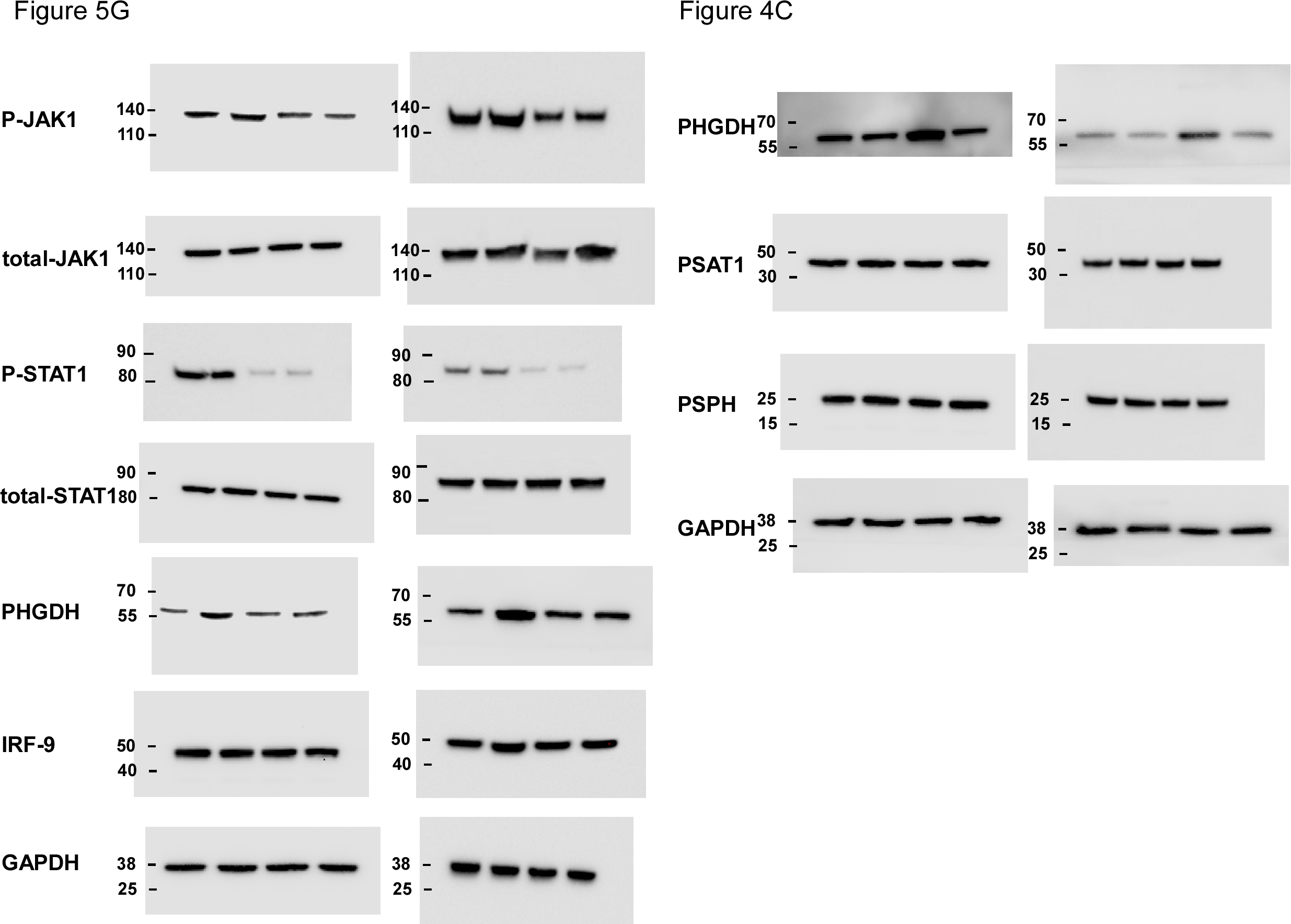

Supplement: Supplementary file 2 — western blots [file 41419_2025_7712_MOESM2_ESM.tif]
